# Neuropsychological Outcomes in Adult Patients and Survivors of COVID-19

**DOI:** 10.3390/pathogens11040465

**Published:** 2022-04-14

**Authors:** Pamela E. May

**Affiliations:** Department of Neurological Sciences, University of Nebraska Medical Center, Omaha, NE 68198-8425, USA; pam.may@unmc.edu

**Keywords:** COVID-19, coronavirus, neuropsychology, cognition

## Abstract

Severe acute respiratory syndrome coronavirus 2 (SARS-CoV-2) is believed to affect central nervous system functions through various indirect, and possibly direct, mechanisms. We are only now beginning to understand the possible effects of the virus on human cognition. This review summarizes extant yet limited literature on clinical neuropsychological findings in adult coronavirus disease 2019 (COVID-19) patients and survivors. Neuropsychological outcomes were often in the form of cognitive screen results, although various studies administered comprehensive batteries. With respect to screens, the Montreal Cognitive Assessment appeared relatively sensitive to cognitive dysfunction associated with COVID-19. Patients and survivors presented with weaknesses on screens and comprehensive batteries, although the pattern of these weaknesses was not specific to etiology. Broadly, weaknesses were suggestive of executive dysfunction, although more than one study did not detect significant impairment. Weaknesses should be interpreted cautiously due to potential confounds/contributing factors (weaknesses may partly reflect psychiatric sequelae; weaknesses may be over-interpreted due to inadequate assessment of premorbid functioning). Studies reported different approaches in defining impairment, likely contributing to variable findings. The current review discusses ongoing efforts to harmonize approaches to evaluating neuropsychological functioning globally, as well as emphasizes taking a comprehensive approach towards understanding how the disease affects cognition.

## 1. Introduction

Survivors of COVID-19 often report subjective, cognitive complaints following infection [1,2], grossly characterized by cognitive inefficiency or “brain fog”. The nature of their objective, neuropsychological presentations are only beginning to be characterized. Risk factors predictive of neuropsychological outcomes post-infection remain under study. The contagiousness and quick rate of severe acute respiratory syndrome coronavirus 2 (SARS-CoV-2) transmission, as well as the significant impact of COVID-19 disease on public health, make the study of COVID-19 on neuropsychological functioning particularly important. 

By means of introduction, the clinical features of COVID-19 are typically classified as mild/asymptomatic, moderate, severe, or critical [3]. While patients with mild symptoms may not need specific interventions, patients with moderate to critical symptoms experience declines in oxygen saturation and require close monitoring [3]. Patients with severe to critical illness require additional oxygen to aid their natural respiratory process and mechanical ventilation, respectively [3]. As such, there are likely effects of respiratory illness or hypoxia on cognition for more progressed disease presentations, particularly in the context of other infectious sequelae (including cerebrovascular and inflammatory changes), advanced age, and pre-existing medical conditions [4]. The effect of hypoxia on cognition can be stated with confidence, as patients have been reported to experience neuropsychological impairment with acute respiratory distress syndrome (ARDS), a possible complication of COVID-19 that leads to low blood oxygen and likely hypoxia. Riordan et al. [5] excellently reviews neuropsychological outcomes associated with acute pulmonary diseases, including ARDS, and noted that delirium is common. Survivors of ARDS may exhibit chronic neuropsychological impairments, including memory dysfunction [5].

SARS-CoV-2 may contribute to cerebrovascular dysfunction, and thus neuropsychological change. Specifically, SARS-CoV-2 binds to a receptor, angiotensin-converting enzyme 2 (ACE-2), that is concentrated in endothelial cells, including cerebral blood vessels [6]. This binding leads to a depletion of ACE-2, causing a cascade of effects that may contribute to endothelial dysfunction and a hypercoagulable state [6], ultimately setting the stage for stroke. A cross-sectional U.K. surveillance study revealed that strokes in COVID-19 patients were usually associated with more progressed illness, including multiple organ failure and severe pneumonia, as well as pre-existing cerebrovascular disease/other risk factors [7]. For COVID-19 patients who sustained stroke, ischemic was the most common stroke type, followed by hemorrhagic [7]. Qureshi et al. [8] reviewed the prevalence of stroke across 54 health care facilities, and reported that acute ischemic stroke was in fact infrequent in patients with COVID-19, yet when it occurred, older adults and African American individuals were relatively at greater risk. 

Further, inflammatory changes are associated with COVID-19, potentially contributing to neurological and neuropsychological symptoms. Per recent reviews [9,10], SARS-CoV-2 appears to overcome the body’s attempts to contain its spread, contributing to a positive feedback loop of continued viral propagation and release of cytokines/chemokines, ultimately leading to the cytokine storm which is characteristic of the disease. Some cytokines may cross the blood brain barrier and activate microglia, increasing inflammatory changes in CNS, and possibly lead to excitotoxicity and neuronal loss. Such changes may contribute to, exacerbate, and/or prolong cognitive symptoms for COVID-19 patients and survivors.

While still under study, the SARS-CoV-2 virus may directly invade the CNS through various routes, including the olfactory and hematogenous routes [11,12]. Defining specific pathways of SARS-CoV-2 will help us to understand possible neurological and cognitive sequelae. Of equal importance, we need to improve our understanding of how COVID-19 may affect neuropsychological functions through other mechanisms, such as fatigue and psychiatric health. The current review aimed to outline neuropsychological weaknesses associated with COVID-19 symptom severity/course, while also provide directions for future studies.

## 2. Methods

Research papers primarily focusing on neuropsychological outcomes associated with COVID-19 were searched under PubMed. The time of publication was limited to 2019 to 1 February 2022. Search criteria terms were “neuropsychological” and “COVID-19” in the paper title or abstract, generating 177 results. Additional papers beyond this search were added based on a manual review of references/papers cited from the PubMed search, as well as a review of recommended, similar articles. Papers that were not available in English or focused on pediatrics or adolescents were not considered. Studies with sample sizes of 10 or less COVID-19 patients or studies without sufficient information on neuropsychological methods/findings were not considered. To reduce variability in methods, the current review was restricted to studies including objective, clinical neuropsychological, or cognitive measures that were administered in person (rather than remotely via computer, tablet, or telephone). A total of 19 neuropsychological research studies met the above criteria and were included in this review. 

## 3. Results: Neuropsychological Outcomes Associated with COVID-19

### 3.1. Neuropsychological Outcomes in Post-Acute Inpatients

Investigations of neuropsychological presentations of inpatients typically involved small samples with minimal neuropsychological testing (namely cognitive screens), given considerations regarding patients’ health and ability to complete testing at bedside. With this context in mind, Beaud et al. [13] reported cognitive findings in an older adult inpatient sample following the critical, acute stage of severe COVID-19. Per performances on cognitive screening (Montreal Cognitive Assessment [MoCA [14]] and Frontal Assessment Battery [FAB [15]]), two cognitive profiles emerged, including (1) normal screen scores with tendency for slightly lower scores on specific executive functioning items/tasks, and (2) mildly to severely low scores across screens, with particularly poor scores on MoCA items assessing executive function, memory, and attention. They reported an association of ICU delirium with poorer MoCA outcomes, but not FAB outcomes. 

Alemanno et al. [16] interestingly evaluated the association of cognitive screening results (MoCA and MMSE [17]) during the subacute phase of infection (approximately 10 days after symptom onset) to different forms of respiratory assistance during the acute phase of infection for inpatients in a COVID-19 rehabilitation unit. Results suggested that patients who required more aggressive or invasive respiratory assistance during the acute phase tended to perform better on cognitive screens during the subacute phase of the disease, relative to patient groups that required less aggressive forms of respiratory assistance. Nevertheless, patients who underwent aggressive respiratory assistance still performed below the cut-off for normal performance on at least the MoCA (M = 21.7, SD = 5.2). Patients who required the most aggressive respiratory assistance tended to be the youngest, and, as such, age was postulated to explain their relatively more preserved cognitive status. They also found that the MoCA appeared more sensitive than the MMSE in detecting cognitive weaknesses in their inpatient sample. 

Pistarini et al. [18] similarly reported greater sensitivity of the MoCA relative to the MMSE for inpatients recovering from COVID-19, as a greater proportion of the subacute sample scored in the impaired range (score < 26) on the MoCA (75%), relative to the MMSE (score of <23.8 adjusted for age and education) (35%). 

Bonizzato et al. [19] assessed change in performance on cognitive screens over time, as patients completed the MoCA and MMSE upon arrival at and discharge from a rehabilitative hospital. The authors reported that about half or more of their sample performed below cut-offs for these screens at first; however, upon discharge (typically about one month later), half or less of the sample scored below these cut-offs. Change in cognitive screen scores over time was not statistically significant. 

A few studies administered detailed neuropsychological tasks beyond the MMSE or MoCA to inpatients recovering from COVID-19. Di Pietro et al. [20] completed a retrospective analysis of neuropsychological performance on a small sample of inpatients undergoing neurological and respiratory rehabilitation. Neuropsychological battery included the MMSE, as well specific measures of immediate memory/attention, verbal and visual memory, visuospatial skill, verbal fluency, problem solving, and other executive functions. Their primary findings were that inpatients with poorer attention tended to have lower functional scores at admission, yet these patients significantly benefited from rehabilitation. In addition, Jaywant et al. [21] administered a brief screen specific to memory and executive functions to inpatients requiring rehabilitation. The greatest areas of deficit were on tasks assessing working memory, divided attention, set-shifting, and processing speed. While they also found deficits in orientation (largely due to incorrectly stating the date or floor number), they opined that this was due to patients’ lengthy hospitalization rather than clinically significant disorientation. Impairments were less observed in the areas of motor speed, delayed memory, or recognition memory. 

A few of the aforementioned studies included follow-up analyses of their patients post-discharge. Alemanno et al. [16] reported that about one month after hospital discharge, a large portion of their patient sample continued to perform below expectations on the MoCA, yet these scores tended to be higher (or better) at discharge relative to admission. Fewer patients performed below expectations on the MMSE at one-month follow-up relative to the MoCA, yet this may have been secondary to the MoCA’s greater sensitivity to cognitive dysfunction. Alternatively, Bonizzato et al. [19] reported that there were no significant changes in performance on MoCA and MMSE scores from admission to three months afterward, although the final sample of patients at the last time point was small (n = 8). Table 1 briefly summarizes reviewed studies’ samples and methods.

### 3.2. Beyond MMSE and MoCA: Neuropsychological Outcomes Following Hospital Discharge

Poletti et al. [22] compared neuropsychological outcomes for COVID-19 patients at one-month, three-months, and six-months points post-discharge (although only a small portion of patients were assessed longitudinally) against patients with depression and healthy controls. Neuropsychological subtests assessing verbal memory, verbal fluency, working memory, attention and processing speed, executive functions, and psychomotor coordination were administered. A global cognitive index score was calculated from these task scores, and this index score did not significantly differ by COVID-19 group (at one-, three-, or six-months post-discharge). Nevertheless, when examining individual cognitive domains at each time point, the percentage of patients without any impairment slightly increased (21%, 25%, to 32%). Further, in a small subsample of patients followed longitudinally, verbal fluency and processing speed tended to improve over time with improvements in mood. The authors noted that COVID-19 patients tended to perform poorer than healthy controls in psychomotor coordination, verbal fluency, executive functions, attention, and processing speed. Patients tended to perform like patients with depression in verbal fluency and executive functions. Further, patients performed like healthy controls in working memory and verbal memory. Findings added that depressive symptoms seemed to best predict cognitive functioning in patients, more so than clinical parameters associated with COVID-19 symptoms (such as duration of hospitalization and ICU stay, medical comorbidities, ventilation use, etc.). The authors emphasized the importance of treating mental health in patients with persistent cognitive weaknesses. While not reviewed extensively here, please see their report on earlier follow-up outcomes [23]. 

Gouraud et al. [24] also focused on associations between neuropsychological performances and psychiatric distress, along with subjective cognitive complaints. They reported that objective neuropsychological test scores were not significantly associated with subjective cognitive complaints, and that subjective cognitive complaints were associated with anxiety and depressive symptoms. Their findings suggested that mental health should be a key area of assessment and management for COVID-19 patients.

Almeria et al. [25] administered a comprehensive neuropsychological battery, including tests of learning/memory, attention, processing speed, executive functions, and aspects of language to patients following discharge. Interestingly, a review of their reported demographically corrected neuropsychological mean scores did not reveal any that fell more than 1 SD below the norm. Ferrucci et al. [26] also used a relatively comprehensive neuropsychological battery, assessing verbal and visual memory, attention, processing speed, working memory, and semantic verbal fluency, and used previously published cut-off scores to detect deficits. Approximately 42% of patients evidenced lower than expected scores on processing speed. Relatively fewer patients (26.3%) showed lower than expected scores on delayed verbal recall. Even smaller proportions evidenced lower than expected scores on other administered tests. Participants with a history of ARDS tended to perform more poorly on a verbal memory task. 

Additional studies went into detail to define neuropsychological impairment for their study. Miskowiak et al. [27] used 0.5 to 1 standard deviation (SD) below the normative mean (based on regression-based formulas on age, sex, and education) as a cut-off for neuropsychological impairment. Using the more conservative cut-off of ≥1 SD below demographically adjusted norms, 11 COVID-19 patients (38% of patients) were reported to evidence “global” cognitive impairment per total score on a cognitive screen (i.e., Screen for Cognitive Impairment in Psychiatry Danish Version; SCIP-D [28]). An additional six patients (21% of patient sample) evidenced “selective” impairment (scores were ≥1 SD below expectations on two or more individual subtests/tests). In contrast, Hellgren et al. [29] considered cognitive performance to be “severely” impaired when a patient scored 2 SD below the normative mean on at least two Repeatable Battery for the Assessment of Neuropsychological Status (RBANS [30]) indices, or 1.5 SD below the mean on at least three RBANS indices. Performance was considered “mildly/moderately” impaired when a patient scored at least 2 SD below the mean on one of the RBANS indices or 1.5 SD below the mean on two RBANS indices. Using these definitions, 46% (16 of 35) of their sample evidenced cognitive impairment, with 6 of these presenting with mild/moderate impairment, and 10 of these presenting with severe impairment. Scores on tests of learning and delayed recall tended to be the most frequent area of impairment. Table 2 briefly summarizes reviewed studies’ samples and methods.

### 3.3. Long-Term Follow-Up in Patients Who Were Not Necessarily Hospitalized

Mattioli et. al. [31] assessed health care workers’ post recovery from mild to moderate COVID-19 illness with a comprehensive neuropsychological evaluation, and compared their findings to a COVID-19 negative group similar in age and sex distribution. Their neuropsychological battery assessed verbal fluency, visuospatial skills, visuospatial memory, verbal memory, reaction time and attention, executive function, and general cognitive status. The authors compared raw scores across neuropsychological tests between post-COVID and COVID-negative patients, and did not detect significant differences. Scores from the MMSE were also normal in both groups. Nevertheless, the authors found significant differences in groups with respect to reported psychiatric symptoms, with the post-COVID-19 group reporting greater anxiety, stress, and depressive symptoms. 

Ferrando et al. [32] assessed global cognitive status (i.e., overall performance on the RBANS), attention, learning/memory, visuospatial skills, psychomotor speed, language, and executive function in post-acute infection patients. Test scores were converted to demographically corrected T-scores per normative data, as well as categorized by “unimpaired” or “extremely low” (i.e., two or more SDs below norm). Performance on the overall RBANS, as well as performances on select RBANS subtests assessing learning, memory, and language, were typically below normative means. Further, 27% of the sample had an “extremely low” score on at least one test. Reported peak COVID-19 symptoms, depressive symptoms, number of medical comorbidities, and self-reported cognitive complaints predicted extremely low neuropsychological test scores. Table 3 briefly summarizes reviewed studies’ samples and methods.

### 3.4. Comparison of Neuropsychological Outcomes by Treatment Setting and COVID-19 Severity

Becker et al. [33] compared the neuropsychological profiles and frequency of impairment of COVID-19 patients who were treated in outpatient, emergency department, and inpatient settings. Patients underwent neuropsychological testing several months after their infection. Neuropsychological test scores were transformed to demographically corrected z-scores. Hospitalized patients were more likely than outpatients to evidence impairments on tests of attention, executive functioning, category fluency, learning, and delayed recall. Patients who were seen in the ED were more likely to evidence impairments on tests of category fluency and learning relative to those seen in an outpatient setting. The authors concluded that hospitalized patients presented with impairments relatively frequently, and that COVID-19 patients seemed to exhibit a profile suggestive of executive dysfunction. 

Similarly, Mattioli et al. [34] examined neuropsychological outcomes of patients with mild to moderate COVID-19 versus patients with severe/critical COVID-19 (ICU cases) around four months after diagnosis. Relative to the mild/moderate group, the severe/critical cases were more likely to be male, older, have fewer years of education, and have diabetes and hypertension. At four months, MMSE scores were within normal limits for all patients, yet the severe/critical group tended to score lower on the MMSE than the mild/moderate group. Raw mean scores of all administered neuropsychological tests were lower in the the severe/critical group relative to the mild/moderate group. Raw test scores were transformed to demographically corrected z-scores, then graded by level of impairment (if z = 0 to 1, then grade = 0; if z = 0 to −1, then grade = 1, etc.). An absolute cognitive index was obtained by summing these graded scores to indicate the level of overall impairment. The severe/critical group had worse cognitive index scores relative to the mild/moderate group. The authors concluded that the severe/critical COVID-19 group appeared to be relatively more vulnerable to developing longstanding cognitive impairment. 

Further, Bungenberg et al. [35] compared neuropsychological outcomes of patients who were hospitalized, relative to those were not hospitalized. Only patients with persisting COVID-19 symptoms were included in their analysis. A comprehensive neuropsychological battery was administered, and scores were transformed using demographically adjusted norms. A score below the sixteenth percentile was considered impaired, with “severe” impairment defined as below the second percentile. While neuropsychological findings were typically within normal limits in both groups, “severe” impairment was present for a minority of patients in the areas of reaction time and phonemic verbal fluency, and less severe impairment was observed in aspects of memory. The hospitalized patients tended to perform worse on the MoCA and on additional tasks of logical reasoning and aspects of verbal learning relative to the non-hospitalized patients. Greater fatigue ratings were associated with poorer scores in attention and reaction time in both groups. Table 4 briefly summarizes reviewed studies’ samples and methods.

### 3.5. COVID-19 Long-Haulers

Few studies have comprehensively examined neuropsychological functioning in patients reporting persistent symptoms despite relatively mild COVID-19 illness at onset. These patients have been known as “long haulers”. Dressing et al. [36] assessed outpatients reporting lasting neurocognitive symptoms > three months post-COVID-19 infection with the MoCA and a comprehensive neuropsychological battery, evaluating demographically corrected scores from tasks of learning/memory, auditory attention, processing speed and executive function, and verbal fluency. Impairment was defined as 1.5 SD below the normative mean. Nearly half of the participants did not present with impairments in this battery, although some exhibited impairments in single domains, most frequently visual memory. The sample’s mean score on the MoCA was considered normal (27 out of 30). 

Apple et al. [37] examined factors that may account for prolonged cognitive difficulties for non-hospitalized patients experiencing mild COVID-19 infection. Healthy controls and patients reporting prolonged cognitive symptoms post-COVID-19 infection underwent comprehensive neuropsychological evaluation. Individuals were classified as having cognitive impairment if they had demographically corrected scores 1 SD or more below the normative mean on one or more tests in two or more cognitive domains. With this in mind, 13 of 22 post-acute COVID-19 patients met the objective criteria for cognitive impairment. Further, relative to the control group, post-acute COVID-19 patients reported a greater median number of risk factors for cognitive impairment relative to controls, per their reported medical, psychiatric, and substance use histories. Nearly 45% of the patient sample reported a delayed onset of persistent cognitive symptoms. Overall, Apple et al.’s findings highlight the complex, multifactorial nature of cognitive complaints in patients post-mild COVID-19. Table 5 briefly summarizes reviewed studies’ samples and methods.

## 4. Discussion

As per the reviewed literature, individuals with COVID-19 are at risk of experiencing cognitive weaknesses following infection, with weaknesses being present for months afterward. This conclusion is strengthened by the use of comparisons against healthy or COVID-negative controls, as well as the use of demographically corrected neuropsychological test scores. Nevertheless, we do not have a strong consensus as to whether there truly has been a “change” in neuropsychological scores for patients at the individual level secondary to COVID-19 infection, given the limited investigation of pre-infection neuropsychological data for comparison or consistent, sound assessment of premorbid functioning. An exception to this statement is work by Douaud et al. [38], who recently reported longitudinal findings on structural neuroimaging and cognitive findings for COVID-19 patients (pre- and post- infection) and controls. The researchers found that COVID-19 patients, relative to controls, evidenced neuroanatomical changes associated with limbic and olfactory cortical systems and a greater reduction in global brain size [38]. The COVID-19 group performed more slowly on tasks of processing speed and executive function post-infection, relative to the baseline assessment [38]. Such longitudinal work is promising for strengthening our understanding of COVID-19 sequelae. 

The data at hand point to possible predictors of neuropsychological dysfunction, yet these need to be assessed more thoroughly and rigorously. While it remains unclear if COVID-19 symptom severity is associated with degree of neuropsychological dysfunction, some findings provide insights. Mattioli et al. [31] opined that the absence of clear neuropsychological dysfunction in their cohort may have been related to the mild to moderate severity of the COVID-19 experienced by most of their participants. This was further strengthened by their follow-up paper [34], in which they reported that neuropsychological impairment was more frequent and prominent in severe to critical COVID-19 patients, relative to mild to moderate COVID-19 patients. There is also data to suggest that olfactory dysfunction associated with COVID-19 is more predictive of cognitive impairment than the severity of acute COVID-19 itself [39]. Further, while also requiring further evaluation, age, and treatment needs (such as hospitalization) may predict degree of neuropsychological dysfunction. Studies assessing younger COVID-19 patients (including Almeria et al. [25]) seemed to experience relatively favorable cognitive outcomes. Findings by Becker et al. [33], Bungenberg [35], and preliminary data per Vergori et al. [40] (not included in this review) reported that neuropsychological impairment was more common or severe in COVID-19 patients who were previously hospitalized versus patients who did not require hospitalization. 

Another key point is that there is no single, clear cognitive profile associated with COVID-19. While attention, processing speed, and executive functions appeared to be most commonly affected in patients with pronounced disease or in patients who were assessed proximal to hospital discharge (consider findings by Beaud et al. [13] and Jaywant et al. [21]), this neuropsychological pattern could be a by-product of the selected test battery. Studies examining patients more distal to time of infection more frequently used comprehensive neuropsychological batteries and reported dysfunction associated with memory, in addition to other areas. While a dysexecutive neuropsychological profile may best characterize COVID-19 findings per review of studies, it is emphasized that this profile is not specific to etiology, and may reflect the complex, multisystemic effects of COVID-19 disease. 

Of the few studies that examined patients across varying cross-sectional time points (i.e., Poletti et al. [22]) or followed patients briefly over time post infection (e.g., Alemanno et al. [16]), there was indication of trends for cognitive recovery. Longitudinal investigation of cognitive trajectories is of great interest, with care taken to avoid practice effects over time (e.g., consideration of alternate test forms). 

While not the focus of this paper, studies variably included assessment of psychiatric symptoms. Of interest, Ferrando et al. [32] reported that depressive symptoms significantly predicted extremely low neuropsychological test scores several months post-infection, raising the question as to what extent cognitive dysfunction reflects aggravated psychiatric symptoms, particularly in adults without history of severe/critical illness. Mattioli et al.’s [31] and Gouraud et al.’s [24] findings also highlighted elevations in psychiatric distress in their patient samples, with the latter study suggesting that psychiatric distress is significantly tied to subjective cognitive complaints. Further, based on limited studies of objective, neuropsychological functioning in long-haul COVID-19 patients, there is a range of those presenting normally on exam as well as those presenting with impairments, raising questions as to what may directly account for these differences.

The current review raises additional questions regarding best practices for neuropsychological assessment and definitions of impairment in COVID-19 research. Per studies on inpatients, it appears that the MoCA has greater sensitivity than the MMSE in detecting cognitive dysfunction associated with COVID-19, holding value for future studies involving screens. These findings are in line with specific investigation of the utility of the MoCA and MMSE in detecting cognitive weaknesses in COVID-19 patients [41]. A few of the reviewed studies created definitions for cognitive impairment (e.g., 1 to 2 SD below normative mean, with differences in how many tests or domains this applied to) when using comprehensive neuropsychological testing. While this nears procedures used in clinical practice, the varying thresholds for cut-offs may contribute to some variability in findings across studies. A consistent definition for impairment, with consideration of premorbid functioning and possible confounds, is critical for comparing findings across studies. Further, performance validity is often not reported in studies, and should be an area more consistently addressed, in order to increase confidence in reported findings.

Researchers are commended for working efficiently to report neuropsychological findings associated with the evolving nature of SARS-CoV-2 and its effects. Nevertheless, it remains too early to report reliable, firm conclusions on the effect of the virus on neuropsychological functioning. Many published studies are limited by small sample sizes or case studies, which remain informative, yet raise questions as to how well findings reliably generalize to larger, more diverse samples. There has been little time to work on harmonizing neuropsychological assessment approaches, with respect to the use of specific tests, timing, and settings of neuropsychological evaluations. The heterogeneity of test selection and methods make it difficult to compare findings across studies. Unfortunately, it is not reasonably valid to compare scores from different tests assessing similar constructs, in the context of slight differences in methods (and how that might impact responses), differences in normative data, and potential differences with respect to cultural and linguistic factors. While use of the widely available cognitive screens, such as the MoCA and MMSE, have led to a common neuropsychological outcome across studies, some studies use differing cut-offs for defining impairment. Further, conclusions regarding specific cognitive profiles or areas of weakness per screening outcomes should be interpreted with caution, as these screens do not thoroughly assess specific cognitive domains, and thus are not sensitive enough to detect specific strengths or weaknesses. Of concern, it is questioned if over-reliance on cognitive screens may lead to inappropriate conclusions of specific areas of deficit associated with COVID-19. There are also cultural and sociodemographic factors that must be considered with the interpretation of findings of common cognitive screens and other neuropsychological tasks across national and international studies. 

At this time, there are no defined standards for assessing neuropsychological functions in patients with COVID-19, at least from a research perspective. In reaction to the growing needs for harmonization of neuropsychological methods and procedures/protocols, the NeuroCOVID International Neuropsychology Taskforce was formed in 2020 [42]. Cysique et al. [42] detailed the intent of the taskforce, with the aims of using similar, harmonized assessment methods to combine data from different sources. Guidelines for harmonization include (1) finding means to adequately assess the consequences of COVID-19, including measuring the breadth and severity of possible neuropsychological weaknesses, differentiating neuropsychological weaknesses from psychological distress, assessing neuropsychological presentations at different phases of COVID-19 disease (acute/infections, subacute, and chronic), and considering premorbid and comorbid effects, performance validity, and other pertinent factors; (2) harmonizing methods that are adaptable to a pandemic lockdown, quarantine status, or hospitalization/alertness status (as applicable to telehealth, computerized, remote, pen/pencil assessments) and consideration of use of screening to comprehensive evaluations; and (3) finding methods and procedures that are appropriate for international purposes, including choosing tests that have cross-cultural validity or are widely available [42]. The authors provide recommendations for varying levels of neuropsychological assessment, from minimal to comprehensive, with the most comprehensive being the closest to clinical practice, along with considerations for assessing additional factors that should be assessed as they may interact with cognitive status.

In closing, the extant, yet small literature on objective, neuropsychological outcomes in COVID-19 patients provides an excellent springboard for future research endeavors. Future studies are encouraged to refer to the excellent guidelines as proposed by Cysique et al. [42] in order to promote harmonization of findings for comparability and assessment of clinically relevant outcomes across the disease trajectory for patients of varying demographics and symptom severity. Furthermore, thorough investigation of neuropsychological effects in a harmonized approach will go a long way in informing clinical care and rehabilitative needs for individuals affected by COVID-19.

## Figures and Tables

**Table 1:** Overview of neuropsychological studies in post-acute inpatients.

Authors, Country	Sample Size (n)	Mean Age (SD), Unless Otherwise Reported	Time of Assessment	Neuropsychological Battery *
Alemanno et al.; Italy	n = 87 during rehabilitation (reduced to n = 56 at 1-month after discharge)	67.2 (12.9) for initial sample	during inpatient rehabilitation, and one month after discharge	Montreal Cognitive Assessment; Mini-Mental State Exam
Beaud et al.; Switzerland	n = 13	64.8 (7.6)	during hospital admission	Montreal Cognitive Assessment; Frontal Assessment Battery
Bonizzato et al.; Italy	n = 12 at admission and discharge; n = 8 at 3-month follow-up	71.3 (10.1)	time of hospital admission, discharge from rehabilitative hospital, and three-month follow-up	Montreal Cognitive Assessment; Mini-Mental State Exam; Additional neuropsychological tests were administered to small subsample (these findings were not reviewed here)
Di Pietro et al.; Italy	n = 12	64.0 (13.7) (demographics reported for subsample of 10)	during hospitalization	Mini-Mental State Exam; Frontal Assessment Battery; forward and backward digit span; Trail Making Test, Parts A and B; story recall; Rey-Osterrieth complex figure; verbal fluency; clock drawing; Tower of London
Jaywant et al.; USA	n = 57	64.5 (13.9)	during inpatient rehabilitation	Brief Memory and Executive Test
Pistarini et al.; Italy	n = 40	64.1 (11.9)	during inpatient rehabilitation	Montreal Cognitive Assessment; Mini-Mental State Exam

* Please refer to original papers for references of neuropsychological measures.

**Table 2:** Overview of neuropsychological studies assessing patients following hospital discharge.

Authors, Country	Sample Size (n)	Mean Age (SD), Unless Otherwise Reported	Time of Assessment	Neuropsychological Battery *
Almeria et al.; Spain	n = 35	47.6 (8.9)	10 to 35 days post hospital discharge	Test de Aprendizaje Verbal España-Complutense; Wechsler Memory Scale –IV Visual Reproduction; Trail Making Test Parts A and B; Digits forward and backwards; Letter and Numbers; Symbol Digit Modalities Test; Stroop; Phonemic and semantic verbal fluency; Boston Naming Test
Ferrucci et al.; Italy	n = 38	53.5 (12.6)	4 to 5 months following hospital discharge	Montreal Cognitive Assessment; Brief Repeatable Battery of Neuropsychological Tests, comprising Selective Reminding Test; 10/36 Spatial Recall Test; Symbol Digit Modalities Test; the Paced Auditory Serial Addition Test; and the Word List Generation Test
Gouraud et al. France	n = 100	Median = 60 (IQR = 49.5–71.5)	1 month following hospital discharge	Semantic Verbal Fluency Test; Digit Symbol Substitution Test; and Mini Mental State Exam
Hellgren et al.; Sweden	n = 35	Median = 59 (IQR = 51–66)	about 5-months following hospital discharge	Repeatable Battery for Assessment of Neuropsychological Status
Miskowiak et al.; Denmark	n = 29 COVID-19 patients; n = 100 healthy controls	56.2 (10.6) for COVID-19 patients 56.0 (6.9) for healthy controls	3 to 4 months following hospital discharge, for patients	Screen for Cognitive Impairment in Psychiatry Danish Version (SCIP-D); Trail Making Test Part B
Poletti et al.; Italy	n = 312 COVID-19 patients (92 evaluated at 1-month follow-up; 122 evaluated at 3-month follow-up; 98 evaluated at 6-month follow-up) n = 165 controls n = 165 inpatients with depression	53.4 (7.5) at 1-month; 53.5 (10.4) at 3-months; 55.0 (9.8) at 6-months 40.6 (11.8) for controls 49.4 (11.2) for patients with depression	1-, 3-, and 6-months post hospital discharge for patients	Brief Assessment of Cognition in Schizophrenia (BACS)

* Please refer to original papers for references of neuropsychological measures.

**Table 3:** Overview of neuropsychological studies assessing long-term follow-up in patients who were not necessarily hospitalized.

Authors, Country	Sample Size (n)	Mean Age (SD), Unless Otherwise Reported	Time of Assessment	Neuropsychological Battery *
Ferrando et al.; USA	n = 60	41.4 (13.5)	6 to 8 months post-acute infection	Test of Premorbid Function; RBANS, Trail Making Test Parts A and B, verbal fluency, and Stroop Color Word Test
Mattioli et. al.; Italy	n = 120 COVID-19 patients; n = 30 COVID-19 negative controls	47.9 (range: 26–65) for COVID-19 patients 45.7 (range: 23–62) for COVID-19 negative controls	4-months post diagnosis	Controlled Oral Word Association, Rey Complex Figure Copy and Recall, California Verbal Learning Test, TEA attention test, Tower of London test, Mini-Mental State Exam

* Please refer to original papers for references of neuropsychological measures.

**Table 4:** Overview of studies comparing neuropsychological outcomes by treatment setting/COVID-19 severity.

Authors, Country	Sample Size (n)	Mean Age (SD), Unless Otherwise Reported	Time of Assessment	Neuropsychological Battery *
Becker et al.; USA	n = 740	M = 49 IQR = 38–59	Mean of 7.6 (2.7) months following diagnosis	Number Span forward and backward; Trail Making Test Part A and B; phonemic and category fluency; Hopkins Verbal Learning Test-Revised
Bungenberg et al.; Germany	n = 21 (previously hospitalized) n = 29 (non-hospitalized)	M (hospitalized) = 57.3, IQR = 52–62 M (non-hospitalized) = 45.6, IQR = 37–56	Median of 29.3 weeks following diagnosis	Montreal Cognitive Assessment; Test of Attentional Performance; Trail Making Test; Verbal Fluency (using either the CERAD-Plus or the Regensburger Wortflüssigkeit-Test); Stroop test variant (Farbe–Wort–Interferenztest); Auditory Verbal Memory Test; Rey–Osterrieth Complex Figure Test or the figure subtest from CERAD-Plus; Boston Naming Test (from CERAD-Plus)
Mattioli et. al.; Italy	n = 215 (163 mild-moderate; 52 severe-critical)	Mild-moderate M = 46.9 (9.4) Severe-critical M = 60 (9.9)	About 4-months following diagnosis	Controlled Oral Word Association; Rey Complex Figure Copy and Recall; Rey Auditory Verbal Learning Test (in severe-critical patients only); California Verbal Learning Test (for mild-moderate patients only); TEA; Tower of London test; Mini-Mental State Exam

* Please refer to original papers for references of neuropsychological measures.

**Table 5:** Overview of neuropsychological studies assessing COVID-19 long-haulers.

Authors, Country	Sample Size (n)	Mean Age (SD), Unless Otherwise Reported	Time of Assessment	Neuropsychological Battery *
Apple et al.; USA	n = 22 patients with post-acute sequelae of COVID-19; n = 10 for cognitive controls	Median = 47.5 for patients (IQR = 38–53) Median = 39 for cognitive controls (IQR = 30–43)	median of 10.1 months from first COVID-19 symptom	California Verbal Learning Test-3; Rey–Osterrieth Complex Figure Test; Delis-Kaplan Executive Function System Trail Making Test, Verbal Fluency, Design Fluency, and Color-Word Interference; Weschler Adult Intelligence Scale-4th edition, Digit Span, Coding, and Symbol Search subtests; Neuropsychological Assessment Battery (NAB) Visual Discrimination; NAB Naming
Dressing et al.; Germany	n = 31	53.6 (12.0)	>3 months post-mild symptoms	Montreal Cognitive Assessment; Hopkins Verbal Learning Test-Revised; Brief Visuospatial Memory Test-Revised; Digit Span; Trail Making Test, Parts A and B; Color Word Interference Test, Symbol Digits Modalities Test; phonemic and categorical verbal fluency

* Please refer to original papers for references of neuropsychological measures.
